# Enteral sesame oil therapeutically relieves disease severity in rat experimental osteoarthritis

**DOI:** 10.3402/fnr.v60.29807

**Published:** 2016-03-30

**Authors:** Dur-Zong Hsu, Pei-Yi Chu, I-Ming Jou

**Affiliations:** Department of Orthopedics, National Cheng Kung University Hospital, Tainan, Taiwan

**Keywords:** osteoarthritis, muscle weakness, oxidative stress, sesame oil, rats

## Abstract

**Background:**

Osteoarthritis (OA) is the most common cause of joint pain, affecting approximately 15% of the population. Recent studies indicate that quadriceps muscle weakness is directly involved in the pathogenesis of OA-associated joint pain. Oxidative stress plays an important role in skeletal muscle dysfunction. Sesame oil is a natural product with excellent antioxidative property. However, whether sesame oil can decrease OA-induced joint pain has never been investigated.

**Objective:**

The aim of the present study was to examine the effect of sesame oil on OA-induced joint pain in rats.

**Design:**

OA-associated joint pain in rats was induced by medial meniscal transection in rats. Sesame oil (0, 1, 2, or 4 ml/kg/day, orally) was given to rats 7 days after OA induction, while the parameters were determined 7 days after sesame oil administration.

**Results:**

Daily sesame oil treatment for 7 days significantly decreased OA-associated joint pain. Sesame oil decreased muscular interleukin-6 and increased citrate synthase activity and myosin heavy chain IIa mRNA expression. Furthermore, sesame oil decreased muscular lipid peroxidation, nuclear Nrf2 protein expression, and reactive oxygen species generations as well as increased glutathione production and glutathione peroxidase activity in OA rats.

**Conclusions:**

Sesame oil may relieve OA-associated joint pain by inhibiting quadriceps muscular oxidative stress, at least partially, in rats.

Osteoarthritis (OA) is the most common cause of joint pain, affecting approximately 15% of the population. Because of its predilection for lower extremity joints, such as the knee and hip, OA is the leading cause of lower extremity disability among older adults and significantly reduces the quality of life for nearly 30 million Americans (1). Currently, OA is considered a major public health problem worldwide. Because there is no current pharmacologic agent to prevent OA progression, the goal of pharmaceutical therapy is to control pain. Acetaminophen and non-steroidal anti-inflammatory drugs (NSAIDs) are commonly used for managing OA pain. However, gastrointestinal and cardiovascular adverse reactions are frequently observed in patients with long-term treatment of NSAIDs (2). Long-term acetaminophen may cause hepatic dysfunction and injury (3).

Quadriceps muscle weakness is one of the important risk factors in the pathogenesis and development of OA pain (4). Historically, muscle weakness is considered a secondary effect in knee OA; however, recent studies indicate that quadriceps muscle weakness may precede the onset of radiographic evidence of OA and pain (5). Initiating OA in the guinea pig is associated with the changes and dysfunction in quadriceps skeletal muscle (6). It is likely that quadriceps muscle weakness is directly involved in the pathogenesis and development of OA-associated pain (7). Therefore, improving quadriceps weakness may be a strategy for preventing OA initiation and development.

Skeletal muscles are composed of the myofilaments actin and myosin (8). Previous studies indicate that alteration and distribution of these myosin heavy chains (MHCs) play a dominant role in muscle strength (9). For example, decreased muscular MHC IIa mRNA and protein expressions are associated with muscle weakness in OA patients (10). Pro-inflammatory cytokine and citrate synthase (CS) also play a crucial role in the loss of muscle mass and strength (11, 12). A correlation between high levels of interleukin (IL)-6 and low muscle mass and strength was found in OA patients (13). Furthermore, CS activity has been used as an indicator of oxidative potential in skeletal muscle (14, 15).

Recently, oxidative stress is believed to be responsible for many tissue changes including the loss of skeletal muscle mass and strength (16). Reactive oxygen species (ROS), such as superoxide anion, hydroxyl radical, and peroxynitrite, are produced under pathophysiologic conditions (17, 18). If oxidants attack continuously, lipid peroxidation (LPO) of cell membranes ensues and results in ultrastructural injury (19). Nuclear factor erythroid 2–related factor 2 (Nrf2) is a transcriptional activator response to oxidative stress (20). Potent free radicals scavenger glutathione (GSH) prevents interactions of reactive intermediates with critical cellular constituents which play an important role in detoxification and cellular defense (21). It is possible that inhibiting muscular oxidative stress can improve quadriceps muscle function and therefore attenuates disease severity of OA.

Sesame oil, a natural product derived from *Sesamum indicum* L., has been reported to have excellent antioxidative property in various disease models (22–24). However, the effect of sesame oil on OA-induced joint pain has never been investigated. The aim of the study was to examine the therapeutic effect of sesame oil on OA-induced joint pain in rats.

## Materials and methods

### Animals

Male SPF Sprague Dawley rats weighing 200–300 g were obtained from our institution's Laboratory Animal Center. They were housed in a room with a 12-h dark/light cycle and central air conditioning (25°C, 70% humidity), and allowed free access to water and rodent diet. The animal care and experimental protocols were in accordance with nationally approved guidelines (permission number 972333).

### OA-induced joint pain in rats

A small incision was made longitudinally down the medial side of the knee and a cauterizer was used to work through both the connective tissue and muscle layers until the medial collateral ligament, anchoring the medial meniscus to the tibial plateau, was identified. The ligament was grasped at the tibial end and cut until fully transected. The ligament was then transected again at the femoral end to remove the portion overlying the meniscus. The meniscus was freed from the fine connective tissue, allowing a full thickness, medial meniscal transection (25).

### Experimental designs

Rats were divided into five groups of five. Group I (Sham group), rats received Sham operation; Group II (OA group), rats received OA induction only; and Group III–V (OS1, OS2, and OS4 groups), rats were ingested with sesame oil (Sigma, St. Louis, MO, USA) (1, 2, or 4 ml/kg/day, respectively) 7 days after OA induction. Hindlimb weight distribution was assessed 7, 10, and 14 days after OA induction. Biomarkers of muscular dysfunction and oxidative stress as well as ROS generations and endogenous antioxidant levels were determined 14 days after Sham or OA operation.

### Hindlimb weight distribution assessment

Hindlimb weight distribution was measured on an incapacitance meter (IITC, Inc., Woodland Hills, CA, USA). In brief, an incapacitance meter consists of two scales and specialized caging to encourage a rearing posture. Weight-distribution imbalance was determined at each time point by using a repeated measures test, with balanced weight distribution represented by a right limb percentage weight near 50% (26).

### Measuring IL-6 levels

IL-6 levels were quantitatively measured using ELISA kits (Duo-Set; R&D Systems Inc., Minneapolis, MN, USA) following the manufacturer's protocol. The protein concentration in tissue homogenate was determined by using protein assay dye (Bio-Rad Laboratories, Hercules, CA, USA).

### Measuring CS activity

Briefly, the homogenates were frozen and thawed four times to disrupt the mitochondria. We added 4 µl of homogenate to 200 µl assay buffer (100 mM Tris buffer, 5 mM 5,5-dithiobis(2-nitrobenzoate), 22.5 mM acetyl-CoA, and 25 mM oxaloacetate, pH 8.35). The rate change in color was monitored at wavelength of 405 nm at 15-sec intervals for a period of 3 min (27).

### Measuring MHC IIa mRNA expression

The total RNA was extracted from the muscular tissue using TRIZOL reagent (Invitrogen). Quantitative real-time polymerase chain reaction (PCR) was performed using SYBR Green and Applied Biosystems StepOne Real-Time PCR system (Life Technologies, Carlsbad, CA, USA). After reverse transcription, quantitative PCR was performed 40 cycles under the following conditions: initialing at 95°C for 10 min, denaturing at 95°C for 15 sec, and annealing at 60°C for 1 min. Primer pairs were designed against MHC IIa (forward 5′-AAGATCAAATCATCAGTGCC-3′; reverse 5′-TCTAGCAGATATGTCTCGATG-3′) and GAPDH (forward 5′-AACATCATCCCTGCCTCTACTG-3′; reverse 5′-CTCCGACGCCTGCTTCAC-3′). The amounts of MHC IIa mRNA expression were normalized with GAPDH mRNA value.

### Measuring LPO levels

Muscle homogenate (200 µl) was taken for LPO measurement by using a commercial assay kit (Lipid Peroxidase Assay Kit; Calbiochem-Novabiochem Co, Darmstadt, Germany) following the manufacturer's instruction, and the spectrophotometer was read at 586 nm.

### Western blotting

Nuclear extraction kit (Sigma, St. Louis, MO, USA) was used to separate nuclear and cytosolic protein. Fifty micrograms of protein was loaded on SDS–PAGE, and then transferred to nitrocellulose sheets (NEN Life Science Products, Inc., Boston, MA, USA). After blocking, the blots were incubated with Nrf2, GPx, or b-actin antibody in 5% non-fat skimmed milk. After washed, the blots were incubated with secondary antibodies conjugated with alkaline phosphatase (Jackson Immuno Research Laboratories, Inc., Philadelphia, PA, USA). Immunoblots were developed using bromochloroindolyl phosphate/nitroblue tetrazolium solution (Kirkegaard and Perry Laboratories, Inc., Baltimore, MD, USA) (28).

### Determining ROS level

The homogenate was centrifuged at 400 g at 4°C for 30 min. Superoxide, peroxynitrite, or hydroxyl radical were measured using a high-performance chemiluminescence (CL) analyzer (CLA-2100; Tohoku Electronic Industrial Co, Ltd, Rifu, Japan). Briefly, 400 µl of tissue homogenate were mixed with 200 µl of phosphate buffer solution in a stainless dish, and then the background CL count was read for 60 sec. One hundred microliters of lucigenin, indoxyl β-D-glucuronide, or luminol (17 mM dissolved in phosphate buffer solution to determine superoxide anion or hydroxyl radical, respectively) was injected into the machine, and the CL was counted. The data were analyzed using Chemiluminescence Analyzer Data Acquisition Software (Tohoku Electronic Industrial Co) (19).

### Measuring GSH levels

Briefly, muscle tissues were homogenized in trichloroacetic acid. After centrifugation, 500 µl of supernatant was added to 2 ml of 0.3 M Na_2_HPO_4_ solution and 200 µl solution of dithiobisnitrobenzoate (in 1% sodium citrate, 0.4 mg/mL). The absorbance at 412 nm was measured (19).

### Statistical analysis

Data were expressed as the means±standard deviation (SD). One-way analysis of variance (ANOVA) followed by student's *t*-test analysis was used to make pairwise comparisons between the groups. Statistical significance was set at *p*<0.05.

## Results

### Effect of sesame oil on disease severity in rat model of OA

**Fig. 1:**
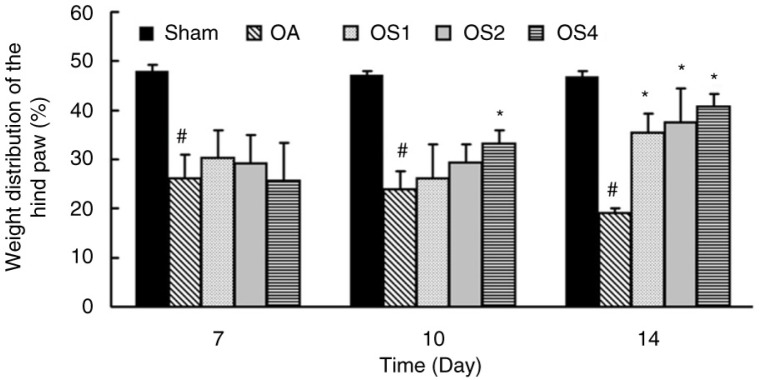
Effect of sesame oil on joint pain in OA. Hindlimb pain was assessed 7, 10, and 14 days after OA induction. Data are means±SD. ^*^P<0.05 compared with the Sham group. ^#^*P*<0.05 compared with the OA group.

To examine the effect of sesame oil on disease severity, weight distribution of the hind paw was assessed in rat model of OA. The weight distribution was significantly decreased at 7, 10, and 14 days in the OA group. However, weight distribution was higher in the OS4 group compared with that in the OA group at 10 days after OA induction. Furthermore, sesame oil at the dose of 1, 2, or 4 ml/kg potently increased the weight distribution compared with the OA group at 14 days after OA induction (Fig. 1).

### Effects of sesame oil on quadriceps muscle dysfunction in rat model of OA

**Fig. 2:**
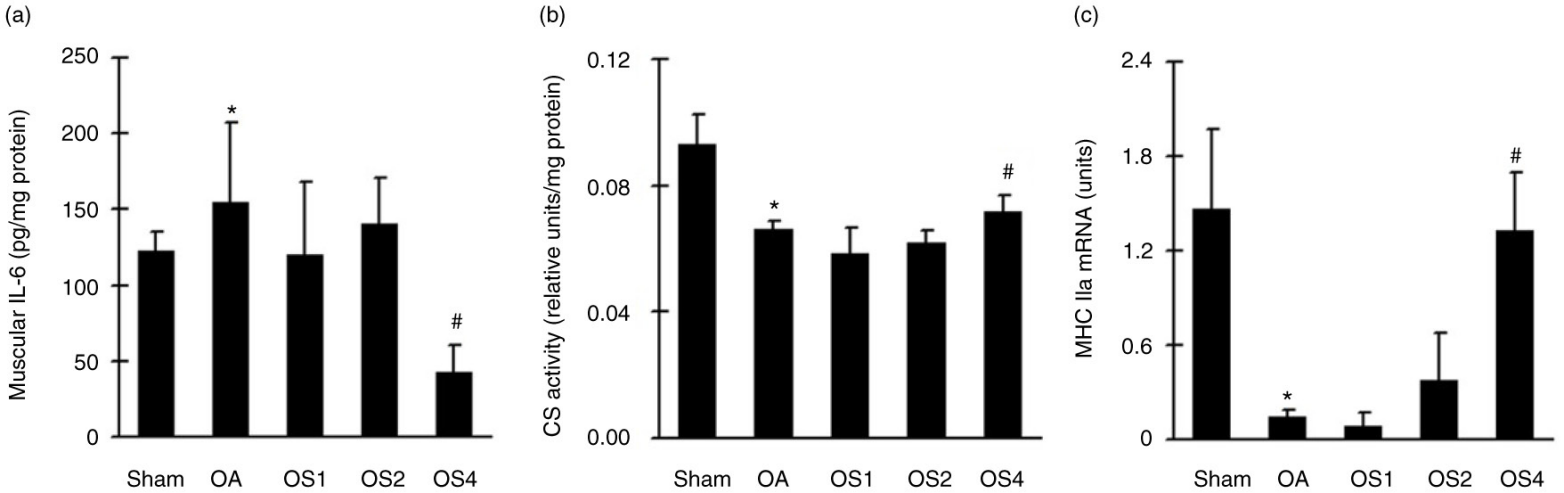
Effect of sesame oil on quadriceps muscle dysfunction in OA. Muscular IL-6 production (a), CS activity (b), and MHC IIa mRNA expression (c) levels were determined 14 days after OA induction. Data are means±SD. ^*^P<0.05 compared with the Sham group. ^#^*P*<0.05 compared with the OA group.

To examine the effects of sesame oil on quadriceps muscle dysfunction, muscular IL-6 production, CS activity, and MHC IIa mRNA levels were determined. Muscular IL-6 production (Fig. 2a) was significantly increased while CS activities (Fig. 2b) and MHC IIa mRNA levels (Fig. 2c) were significantly decreased in OA rats. However, sesame oil at the dose of 4 ml/kg significantly decreased IL-6 levels and increased CS activity and MHC IIa RNA expression compared with the OA group (Fig. 2).

### Effects of sesame oil on quadriceps muscle oxidative stress in rat model of OA

**Fig. 3:**
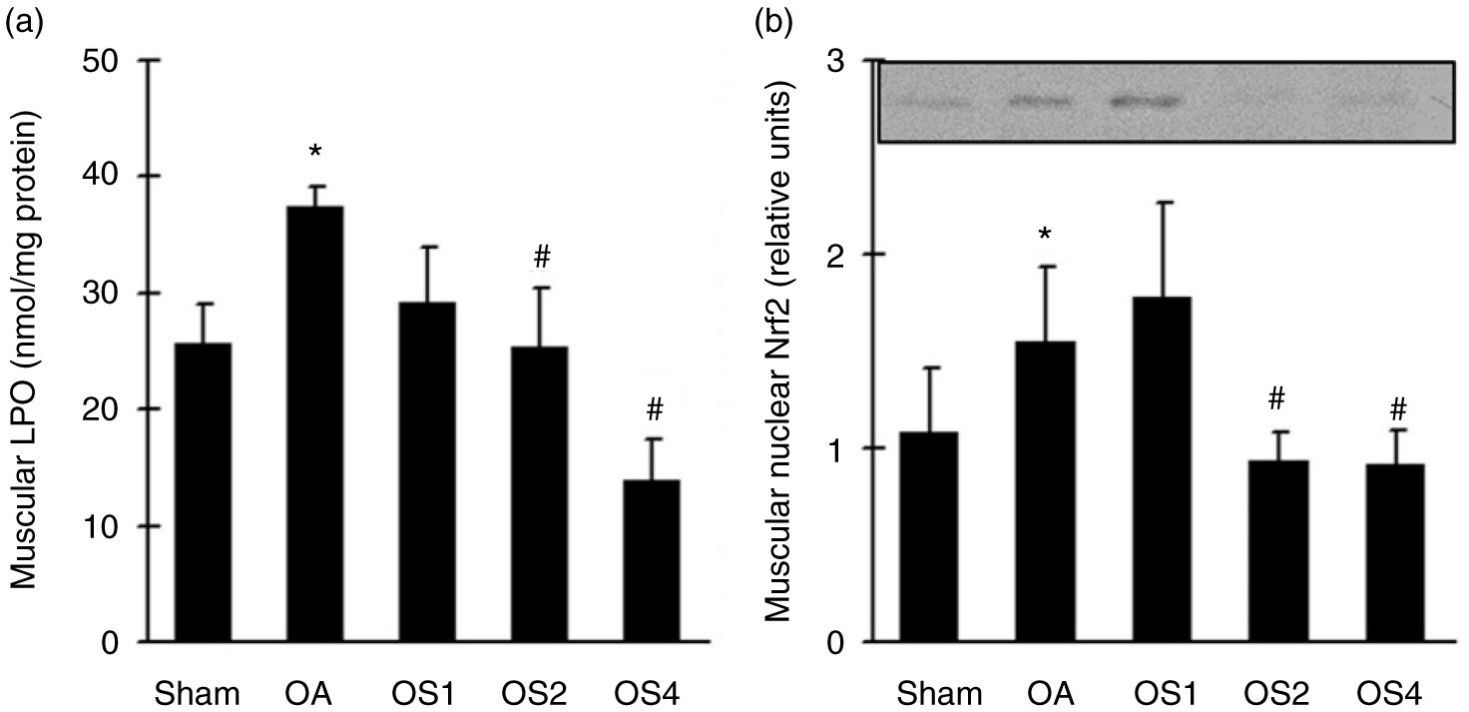
Effect of sesame oil on muscular oxidative stress in OA. Muscular LPO levels (a) and nuclear Nrf2 expression (b) were determined 14 days after OA induction. Data are means±SD. ^*^P<0.05 compared with the Sham group. ^#^*P*<0.05 compared with the OA group.

To examine the involvement of oxidative stress in sesame oil–associated muscular dysfunction attenuation, oxidative stress markers were determined. Muscular LPO (Fig. 3a) and nuclear Nrf2 expression (Fig. 3b) were significantly increased in OA group compared with the control group. Sesame oil significantly decreased LPO level and nuclear Nrf2 expression in OS2 and OS4 groups compared with that in the OA group (Fig. 3).

### Effects of sesame oil on muscular ROS generation and endogenous antioxidant production in rat model of OA

**Fig. 4:**
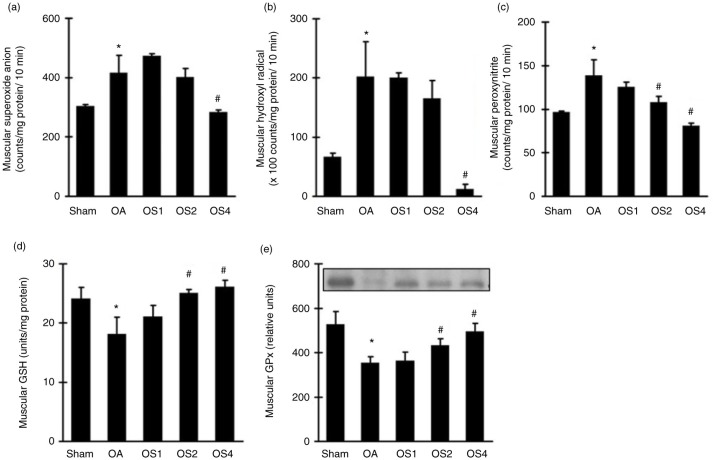
Effect of sesame oil on muscular ROS generation and endogenous antioxidant in OA. Muscular superoxide anion (a), hydroxyl radical (b), and peroxynitrite (c) generations and GSH (d) and GPx (e) levels were determined 14 days after OA induction. Data are means±SD. ^*^P<0.05 compared with the Sham group. ^#^*P*<0.05 compared with the OA group.

To examine the mechanism involved in sesame oil–associated antioxidative effect in quadriceps muscle, ROS, including superoxide anion, hydroxyl radical, and peroxynitrite, as well as endogenous antioxidant GSH and GPx were determined. Muscular superoxide anion (Fig. 4a), hydroxyl radical (Fig. 4b), and peroxynitrite (Fig. 4c) counts were significantly increased 14 days after OA induction. Sesame oil potently decreased the generation of these three ROS in a dose-dependent manner compared with the OA group (Fig. 4). On the contrary, both GSH level (Fig. 4d) and GPx expression (Fig. 4e) were significantly increased in the OS4 group.

## Discussion

For the first time, we demonstrated that sesame oil attenuates OA-associated joint pain in rats. Furthermore, sesame oil improved quadriceps muscle dysfunction, oxidative stress, ROS generation, and increased endogenous antioxidant levels in the rat OA model. We suggested that sesame oil may decrease OA joint pain by inhibiting oxidative stress–associated quadriceps muscle dysfunction.

Sesame oil showed an attenuation of quadriceps muscle dysfunction in OA rats. Both overproduction of IL-6 and decreased CS activity are associated with lower muscle strength and muscle weakness in various animal disease models (29, 30). MHC typing change is one of the major causes of muscle weakness in various pathogenic situations (25, 31). Decreased MHC IIa fiber is associated with lower muscle strength/muscle weakness in the quadriceps in patients with OA (32). In the present study, sesame oil significantly reversed muscular dysfunction and increased MHC IIa gene expression. It is likely that enhancing MHC IIa gene expression may be involved in sesame oil–exerted attenuation of muscular dysfunction, at least partially.

Sesame oil may improve quadriceps muscle dysfunction by inhibiting muscular oxidative stress during the initiation of OA. Genetic evidence has shown that increased oxidative stress in skeletal muscle is sufficient to induce muscle atrophy (33, 34). Elevated ROS generation can contribute to muscle dysfunction by oxidative damage, degradating contractile proteins, or activating calpain and ubiquitin proteolytic systems (35, 36). Overproduction of ROS alters the fiber type and muscle function by regulating MHC gene expression (37). In addition, inhibiting endogenous antioxidant expression in mice results in significant loss of skeletal muscle mass and muscle weakness (16). In the present study, sesame oil decreased muscular LPO and nuclear Nrf2 expression, and muscular ROS generations in experimental OA. Furthermore, GSH and its biosynthesis enzyme GPx levels were increased by sesame oil in experimental OA. We suggest that decreasing muscular oxidative stress is associated with sesame oil–exerted inhibition of OA initiation. In addition, the decrease in ROS generation and the increase in endogenous antioxidant may be associated with sesame oil–exerted antioxidative effect in OA.

Sesame oil may have the potential in modulating OA severity in patients. Selective atrophy of fast-twitch (type 2) fibers might reflect pain-related immobilization of the affected limb, whereas changes such as neurogenic muscular atrophy, muscle fiber degeneration, and regeneration may contribute as cofactors in the development or progression of OA (38). Strengthening of the quadriceps muscles during the initial stages of OA has been reported to have an important physiotherapeutic effect (32). In the present study, sesame oil may decrease joint pain by improving oxidative stress–associated muscle dysfunction.

Sesame oil is rich with antioxidative lignans, including sesamin, sesamol, sesamolin, sesaminol, and sesamolinol. These lignans are suggested to be the most important and effective ingredients in sesame oil against various diseases (39). Therefore, sesame lignans may be the active ingredients in treating OA. However, more studies will be needed to confirm this. We concluded that sesame oil may improve quadriceps muscle by inhibiting oxidative stress in rat model of OA. Furthermore, inhibiting muscular oxidative stress may be beneficial in preventing the initiation and the development of OA.
